# The Role of BRCT Domain from LmjPES in *Leishmania major* Pathogenesis

**DOI:** 10.3390/biom15081191

**Published:** 2025-08-19

**Authors:** Esther Larrea, José Peña-Guerrero, Celia Fernández-Rubio, Aroia Burguete-Mikeo, Elizabeth Guruceaga, Paul Nguewa

**Affiliations:** 1 Unit of Translational Medicine, IdiSNA (Navarra Institute for Health Research), University of Navarra, 31009 Pamplona, Navarra, Spain; elarrea@unav.es; 2 Department of Microbiology and Parasitology, IdiSNA (Navarra Institute for Health Research), University of Navarra, 31009 Pamplona, Navarra, Spain; 3 Bioinformatics Platform, Center for Applied Medical Research, IdiSNA (Navarra Institute for Health Research), University of Navarra, 31009 Pamplona, Navarra, Spain; eguruce@unav.es

**Keywords:** BRCT, *Leishmania*, LmjPES, metabolic pathway, overexpression, parasite, pLEXSY, ribosomal biogenesis, RNAseq

## Abstract

Leishmaniasis is caused by protozoan parasites from the genus *Leishmania* and remains one of the major threats to global health, impacting millions of people worldwide as well as animals including dogs. Several treatments have been used for managing leishmaniasis; nevertheless, drug resistance has emerged as an important obstacle to disease control. Therefore, there is an urgent need to discover new therapeutic targets. The aim of this work was to study the role played by the breast cancer associated 1 C-terminal (BRCT) domain from LmjPES protein (Pescadillo ribosomal biogenesis factor) in *Leishmania major*‘s pathogenesis through the construction of novel genomic tools. For this purpose, *Leishmania* integrative plasmids that were able to express the *BRCT* domain from *LmjPES* and a hypothetical defective *LmjPES* lacking this *BRCT* domain were constructed. It was observed that the overexpression of the aforementioned *BRCT* domain in *L*. *major* dysregulated the mRNA expression of 152 genes (95 up-regulated and 57 down-regulated) in respect to control parasites. Furthermore, clustering studies of these altered genes revealed an enrichment in genes related to metabolic processes, transporter activity, response to stimuli, and protein folding, which are categories described to be associated with the metacyclogenesis process and parasite survival. Interestingly, these genes reached normal levels of expression in parasites transfected with a defective *LmjPES* (a mutated gene lacking the coding sequence of the *BRCT* domain). In addition, it was found that the footpad of mice inoculated with *LmjPES BRCT-*overexpressing parasites had significantly greater inflammation compared to the size of the footpad of animals infected with the control parasites. Based on all these results, it was suggested that the BRCT domain from LmjPES might play a role in *L*. *major*‘s infection process and pathogenesis.

## 1. Introduction

Protozoan parasites belonging to the genus *Leishmania* have been described as the causative agents of leishmaniasis [1]. At least 20 different *Leishmania* spp. are responsible for a variety of clinical manifestations ranging from self-healing skin ulcers (CL) to life-threatening visceral diseases (VL). Leishmaniasis is endemic in around 100 countries, with one billion people at risk. The World Health Organization (WHO) estimated 50,000–90,000 new cases of VL (but only 25–45% of these are registered) per year and 600,000–1 million new cases of CL annually (with around 200,000 reported to the WHO). These data represent cases of human disease. Currently, leishmaniasis is increasingly recognized as a One Health issue. Animals are also infected including dogs, the most important reservoirs of *Leishmania infantum* in Europe [2].

After the bite of infected female phlebotomine sand flies, neutrophils and monocytes migrate to the site of infection. Neutrophils further entrap the parasites [3,4]. Interestingly, during the early stages of the infection, no remarkable changes take place in the epidermis, although it is known that the local microenvironment plays a crucial role in further immune response development [5]. Afterwards, *Leishmania* infection progresses to a silent stage in a few weeks to months, and parasites proliferate without causing any apparent pathology. The silent phase ends with extensive inflammation, with the infiltration of eosinophils, macrophages, and neutrophils, and lesion formation at the inoculation site [6]. Interestingly, the highest burden of parasites is observed just prior to lesion development, suggesting that the effects of the immune response for parasite clearance are the main factors leading to ulcerations and tissue damage [6,7,8].

The life cycle of *Leishmania* parasites consists of two stages with different morphologies. The parasite has a non-motile intracellular amastigote form in the initial stage, which can be detected in mammalian hosts’ phagocytes and circulatory systems. In the second stage, it has a promastigote extracellular elongated and flagellated form, which can be seen in the gut of infected female sand flies [9]. The conversion from non-infective procyclic to infective metacyclic promastigotes is related to biochemical and molecular changes that are not currently well known [10].

The complexity of the *Leishmania* life cycle is partly responsible for the lack of an effective vaccine available against this disease. The control of the parasite relies on treatments that are unfortunately associated with toxicity, difficult to administer, have high costs, or demonstrate drug resistance [11,12]. Therefore, there is a need to investigate new therapeutic targets. One of the most important advancements in the field of leishmanicidal drug development took place decades ago, with the sequencing of the complete genome of *L*. *major* [13], which enabled researchers to search for potential drug targets.

Recently, the homologue of the human oncogene *PES1* (*pescadillo ribosomal biogenesis factor 1*) in *L*. *major* (*LmjPES*) was identified. This was the first study to suggest that this gene is involved in leishmaniasis pathology [14]. Interestingly, the gene expression levels in *L*. *major* parasites dramatically increased in the metacyclic stage (more virulent, infectious, and disease-inducing forms of *Leishmania*) compared to the procyclic stage (with forms exhibiting a low virulence). Therefore, *LmjPES*-overexpressing parasites showed higher in vitro infections as well as increased and faster footpad inflammation in BALB/c mice compared to non-overexpressing parasites. Furthermore, in vivo infection with *LmjPES*-overexpressing parasites correlated with a significantly greater lesion size, supporting the role of LmjPES in *Leishmania* virulence [14]. In mammals, PES1 is known to participate in biological processes such as ribosomal biogenesis [15] and cell growth [16]. Additionally, PES1 is implicated in pathological processes like tumor development [17]. This protein encloses a breast cancer associated 1 (BRCA1) C-terminal (BRCT) domain that has been reported in proteins related to cell cycle regulation and DNA repair [18,19]. It has been described that the accuracy of this domain plays an important role in cell homeostasis and regulation [20,21]. Several investigations have shown that the BRCT domain from PES protein displays important roles in biological cellular processes [15,22]. In *Leishmania*, a BRCT domain in LmjPES protein (LmjPES BRCT) was identified [23]. It was observed that the expression of the *BRCT* domain from *LmjPES* in mammal cells induced both higher survival and replication rates [24]. Moreover, mammal cells expressing this *LmjPES BRCT* domain were more resistant to genotoxic drugs [24]. In this work, the goal was to further analyze the role of the LmjPES BRCT domain in *Leishmania major*‘s biology and pathogenesis.

## 2. Materials and Methods

### 2.1. Plasmid Construction

For this study, two expression vectors were performed as follows. One contained the *LmjPES BRCT* domain and the other one contained *LmjPES ∆BRCT* sequence.

The *BRCT* domain sequence from *LmjPES* gene [23,24] was inserted in the pLEXSY-Hyg expression vector (Jena Bioscience, Jena, Germany) using the In-fusion cloning kit (Clontech, Montain View, CA, USA). After extracting *L*. *major*‘s total genomic DNA following a stablished protocol [25], *LmjPES BRCT* sequence was amplified through PCR using primers designed with the In-Fusion cloning software version PR133833 (Takara, Shiga, Japan). The sense primer (BRCTF1, Table 1) included the sequence from the start of this domain, a start codon (*ATG*), a kozak sequence (*ACACC*), and the sequence of the end of Nco I (New England Biolabs, Ipswich, MA, USA) digested pLEXSY-Hyg vector. The antisense primer (BRCTF2, Table 1) included the sequence from the end of this *BRCT* domain and the sequence of the end of Kpn I (New England Biolabs) digested pLEXSY-Hyg vector. PCR product size was checked by gel electrophoresis and ligated into the previous Nco I and Kpn I digested pLEXSY-Hyg vector, obtaining the pLEXSY-*LmjPES BRCT* plasmid.

*LmjPES ∆BRCT* sequence was achieved with two sets of PCR reactions using the previously described and constructed pLEXSY-Hyg-*lmjPES* vector as template [13]. *PES∆BRCTF1* and *PES∆BRCTR2* primers (Table 1) were designed to cover the beginning of *LmjPES* coding sequence to the first codon of *BRCT* domain, whereas *PES∆BRCTF3* and *PES∆BRCTR4* primers (Table 1) covered the end of *BRCT* domain to the end of *LmjPES* coding sequence. Since *PES∆BRCTF3* oligo was designed to be complementary with the end of *PES∆BRCTR2* oligo, the reaction products were joined by a third PCR reaction, using *PES∆BRCTF1* and *PES∆BRCTR4* primers. This final PCR product was cut (Nco I and Kpn I) and then ligated into the previous Nco I and Kpn I digested pLEXSY-Hyg vector using T4 DNA ligase (Invitrogen, Vilnius, Lithuania), obtaining the pLEXSY-*LmjPES* ∆*BRCT* plasmid.

The presence of the inserts in the vectors was checked by PCR, and the sequence was verified by DNA sequencing carried out at CIMA LAB Diagnostic (Pamplona, Spain).

### 2.2. Cell Culture Conditions

*L*. *major* (Lv39c5) promastigotes were grown with agitation at 26 °C in supplemented M199 medium. Prior to in vivo infectivity assays, promastigotes were maintained under agitation in Schneider’s medium (Gibco Laboratories, Grand Islands, NE, USA) supplemented with 10% (vol/vol) heat-inactivated fetal bovine serum (FBS; Gibco Laboratories, Grand Islands, NE, USA), 50 U/mL penicillin, and 50 µg/mL streptomycin (Sigma-Aldrich, St. Louis, MO, USA). In addition, the following cell lines were grown as mentioned below: *L*. *major*–*LmjPES BRCT#54*, *L*. *major*–*LmjPES BRCT#55*, *L*. *major*–*LmjPES BRCT#56*, *L*. *major*–*LmjPES ∆BRCT#1*, *L*. *major*–*LmjPES ∆BRCT#2*, *L*. *major*–*LmjPES ∆BRCT#3*, *L*. *major*–MC*#4*, *L*. *major*–MC*#5,* and *L*. *major*–MC*#6*. Then, metacyclic *L*. *major* promastigotes isolated by the peanut agglutinin method [26] were collected and used for in vivo experiments.

### 2.3. Generation of the Transgenic L. major Cell Lines

A total of 10^8^ log-phase *L*. *major* parasites were transfected by electroporation with the Swa I (New England Biolabs, Ipswich, MA, USA) digested pLEXSY-*LmjPES BRCT*, pLEXSY-*LmjPES* ∆*BRCT,* or pLEXSY-Hyg plasmids [27]. Transfected colonies were isolated from M199 plates supplemented with Agar Noble (Sigma-Aldrich, St. Louis, MO, USA) and 100 µg/mL of hygromicin B Gold (InvivoGen Europe, Toulouse, France), and later, they grew in complete Schneider’s medium plus hygromicin B Gold. We obtained several overexpressing cell lines. We selected and used the following three cell lines: *L*. *major*–*LmjPES BRCT#54*, *L*. *major*–*LmjPES BRCT#55,* and *L*. *major*–*LmjPES BRCT#56* (parasites transfected with Swa I digested pLEXSY-*LmjPES BRCT* plasmid); three additional cell lines: *L*. *major*–*LmjPES ∆BRCT#1*, *L*. *major*–*LmjPES ∆BRCT#2,* and *L*. *major*–*LmjPES ∆BRCT#3* (parasites transfected with Swa I digested pLEXSY-*LmjPES ∆BRCT* plasmid); and three control cell lines: *L*. *major*–MC*#4*, *L*. *major*–MC*#5,* and *L*. *major*–MC*#6* (parasites transfected with Swa I digested pLEXSY-Hyg plasmid).

### 2.4. RNA-Sequencing Analysis

Total RNA from parasites was extracted using the automated Maxwell system (Promega, Madrid, Spain). Then, RNA quality control was carried out to verify the required standards for RNA quantity and integrity. The samples were shipped on dry ice to Macrogen Company (Seoul, Republic of Korea), where they were sequenced. RNA-seq was performed using Illumina platform following a configuration of paired-end reads of 100 base pairs. RNA sequencing data analysis was performed using the following workflow: (1) the quality of the samples was verified using FastQC software version 0.11.8 (https://www.bioinformatics.babraham.ac.uk/projects/fastqc/) (accessed on 15 November 2022) and the trimming of the reads was performed using trimmomatic [28]; (2) alignment against the *Leishmania major* reference genome (ASM272v2.45) was performed using STAR [29]; (3) gene expression quantification using read counts of exonic gene regions was carried out with featureCounts [30]; (4) the gene annotation reference was Ensembl Bacteria v55 [31]; and (5) differential expression statistical analysis was performed using R/Bioconductor [32]. Data are publicly available in GEO database with the accession number GSE234048.

First, gene expression data were normalized with edgeR [33] and voom [34]. After quality assessment and outlier detection using R/Bioconductor [32], a filtering process was performed. Genes with read counts lower than 4 in more than 50% of the samples for all the studied conditions were considered as not expressed in the experiment under study. LIMMA (Linear Models for Microarray Data) [34] was used to identify genes with significant differential expressions between experimental conditions. Genes were selected as differentially expressed using a B cut off of B > 0. Further functional and clustering analyses were performed and graphical representations were created using R/Bioconductor [32], clusterProfiler [35], and functional annotation databases [12,36].

### 2.5. Quantitative Real Time-PCR (qPCR)

Total RNA was extracted from a culture of 10 × 10^6^ parasites in exponential growth phase using TRIZOL reagent (Sigma Aldrich, St. Louis, MO, USA) and subsequently quantified in a NanoDrop spectrophotometer (Thermo Scientific, Waltham, MA USA). Then, one µg of RNA was treated with Ambion DNA-free Kit (Invitrogen, Waltham, MA, USA) and retrotranscribed with SUPERscript II Reverse Transcriptase (Invitrogen, Waltham, MA, USA) at 42 °C for one hour. The obtained cDNA was used for the qPCR assays using a 7500 real-time PCR system (Applied Biosystems, Foster City, CA, USA), 96-well plates (Applied Biosystems, Foster City, CA, USA), and SYBR Green PCR master mix (Applied Biosystems, Foster City, CA, USA). The sequence of primers used in this study is shown in Table 2, and all qPCR tests were performed at 60 °C (primer annealing temperature). To monitor the specificity, the final PCR products were analyzed by melting curves and electrophoresis gel. The PCR efficiency was in the range of 95–105% for all genes analyzed. The amount of each transcript was expressed relative to the reference gene *GAPDH* as 2^∆Cq^, where ∆Cq represents the difference in quantification cycle between the control *GAPDH* and target genes. Then, those data were normalized to the control mRNA expression values.

### 2.6. In Vivo Infection and Evaluation

Female BALB/c mice were purchased from Harlan Interfauna Ibérica S.A. (Barcelona, Spain). All the procedures involving animals were approved by the Animal Care Ethics Commission of the University of Navarra (approval number: 086-20; 30 March 2021).

Eighteen BALB/c mice were divided into three groups (6 per group). Depending on the *Leishmania* cell lines used for the challenge, the groups of animals were named as follows: *L*. *major*–*LmjPES BRCT* (mice infected with *L*. *major*–*LmjPES BRCT* cell lines), *L*. *major*–MC (animals infected with *L*. *major*–MC control cell lines), or sterile PBS (uninfected mice). The subcutaneous inoculation was performed in the right hind footpad using an insulin syringe. The infection process was realized three times in successive weeks, using 500 metacyclic promastigotes (PNA[-]) dissolved in PBS [37]. The uninfected animals were injected with equal sterile PBS solutions. Footpad swelling was measured weekly with a digital caliper, and the “net swelling” was determined as the difference between the infected (right) and non-infected (left) footpad of each mouse. All animals were euthanized five weeks after the last inoculation.

### 2.7. Hematoxylin and Eosin Staining

Hematoxylin and eosin analyses were performed as previously described [37]. Briefly, footpads were formalin-fixed, decalcified in Osteosoft solution (Merck Millipore, Burlington, MA, USA; 1017281000) for 72 h, paraffin-embedded, and cut into 3 µm thick sections. Later, the sections were stained with hematoxylin and eosin.

### 2.8. Statistical Analysis

Statistical studies were performed using the GraphPad Prism software (version 5.0). Normality was assessed with Shapiro–Wilk W test. A two-sided unpaired Student’s *t*-test or Mann–Whitney U-test was used according to sample distribution. RNA-seq statistical tests were performed using R/Bioconductor as described above. Data are presented as means ± SD. *p*-values < 0.05 were considered statistically significant. The statistical significance was also indicated by * for *p* < 0.05; ** for *p* < 0.01; and “ns” for non-significant differences.

## 3. Results

### 3.1. Generation of L. major Parasites Exhibiting a Constitutive Overexpression of BRCT Domain from LmjPES (LmjPES BRCT) or an Expression of LmjPES Lacking BRCT (LmjPES ΔBRCT)

**Figure 1:**
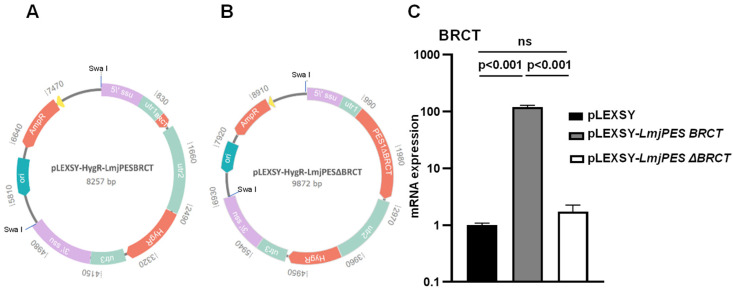
Constructed plasmids and *BRCT* mRNA expression levels. (**A**) pLEXSY-*LmjPES BRCT* construction. (**B**) pLEXSY-*LmjPES ∆BRCT*. 5′ ssu and 3′ ssu: Plasmid regions for genomic integration in the parasite chromosome. Utr1, 2, and 3: Untranslated regions for expression enhancement. Hyg: Hygromycin resistance marker gene for selection in *Leishmania*. Ori: Origin of replication. AmpR: Ampicillin resistance marker gene for selection in bacteria. Yellow: AmpR promoter, The dashed line indicates the number of base pairs mentioned within the plasmid sequence. (**C**) mRNA expression levels of *LmjPES BRCT* domain in several cell lines; *L*. *major* transfected with DNA expression cassette containing the hygromycin resistance sequence and either *LmjPES BRCT* insert *(L*. *major*–*LmjPES BRCT#54*, *#55,* and *#56* cell lines) or *LmjPES ∆BRCT* insert (*L*. *major*–*LmjPES ∆BRCT #1*, *#2,* and *#3* cell lines) or without insert (pLEXSY) (*L*. *major*–MC*#4*, *#5,* and *#6* control cell lines*)*. Data are represented as means of triplicates of each cell line (±SD). *p*-values < 0.05 were considered statistically significant.

In order to understand the implications of the BRCT domain from LmjPES on *L*. *major*‘s biology, we decided to overexpress this domain (LmjPES BRCT). In addition, by using an integrative expression vector, the expression levels of a mutated gene (lacking the coding sequence of the *BRCT* domain) were also assessed. The corresponding constructed plasmids are represented in Figure 1A,B. After *L*. *major*‘s transfection with the SwaI-linearized DNA expression cassette containing the hygromycin resistance sequence and either the *LmjPES BRCT* or *LmjPES ∆BRCT* inserts, or without an insert, several cell lines were obtained. In this work, we present the results corresponding to those cell lines with the highest mRNA expression levels of the *BRCT* domain. As shown in Figure 1C, fold changes of 119.09 ± 9.06 (mean ± SD) for the *LmjPES BRCT*-overexpressing cell lines (*L*. *major*–*LmjPES BRCT#54*, *#55,* and *#56* harboring pLEXSY-*LmjPES BRCT*) and 1.73 ± 0.52 for the *LmjPES ∆BRCT*-expressing cell lines (*L*. *major*–*LmjPES ∆BRCT #1*, *#2,* and *#3* containing pLEXSY-*LmjPES ∆BRCT*) with respect to the control cell lines (*L*. *major*–MC*#4*, *#5,* and *#6)* were registered.

### 3.2. BRCT Domain from LmjPES May Regulate Genes Involved in Metabolic Pathways, Transporter Activity, and Protein Folding

**Figure 2:**
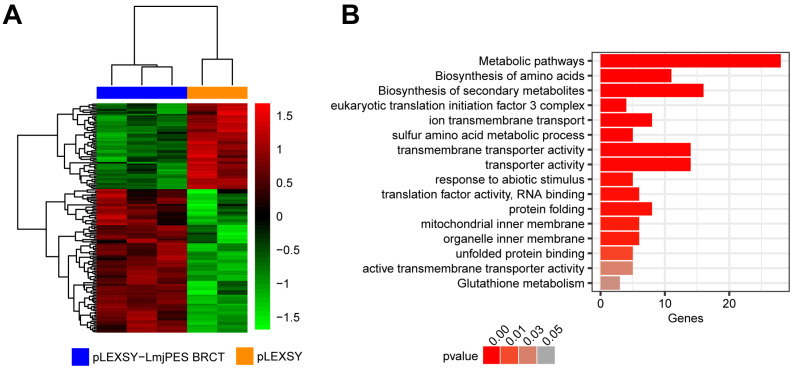
RNA sequencing analysis of *LmjPES BRCT*-overexpressing *L*. *major*. (**A**) Heatmap of 152 dysregulated genes in *LmjPES BRCT*-overexpressing (pLEXSY-LmjPES BRCT) *L*. *major (L*. *major*–*LmjPES BRCT#54*, *#55,* and *#56* cell lines) with respect to control (pLEXSY) parasites (*L*. *major*–MC*#4* and *#5* control cell lines*)*. (**B**) Bar plot of GO and KEGG categories enriched by altered genes observed in the RNAseq analysis.

In a previous work, we described that the expression of the *BRCT* domain from *LmjPES* (the *LmjPES BRCT* domain) in HEK293T and NIH/3T3 cell lines altered the expression of proliferation-, survival- and chemoresistance-related genes in those mammalian cells [24]. The current study aimed to further characterize the biological implications of the *LmjPES BRCT* domain in the parasite. Therefore, high-throughput RNA sequencing of *L*. *major* overexpressing the *LmjPES BRCT* domain *(L*. *major*–*LmjPES BRCT#54*, *#55,* and *#56* cell lines) versus the control parasites (*L*. *major*–MC*#4* and *#5* control cell lines*)* was performed. The data analysis, based on a B statistic cut-off >0, revealed 152 altered (95 up-regulated and 57 down-regulated) gene mRNA levels in *LmjPES BRCT*-overexpressing parasites with respect to the control parasites (Figure 2A and Supplementary Table S1). In addition, functional and clustering analyses of these dysregulated genes were performed using the Gene Ontology (GO) and KEEG (Kyoto Encyclopedia of Genes and Genomes) databases. We mainly found an enrichment in categories related to metabolic processes, transporter activity, response to stimuli, and protein folding (Figure 2B). The aforementioned categories had been described to be involved in the metacyclogenesis process [38] and parasite survival [39].

**Figure 3:**
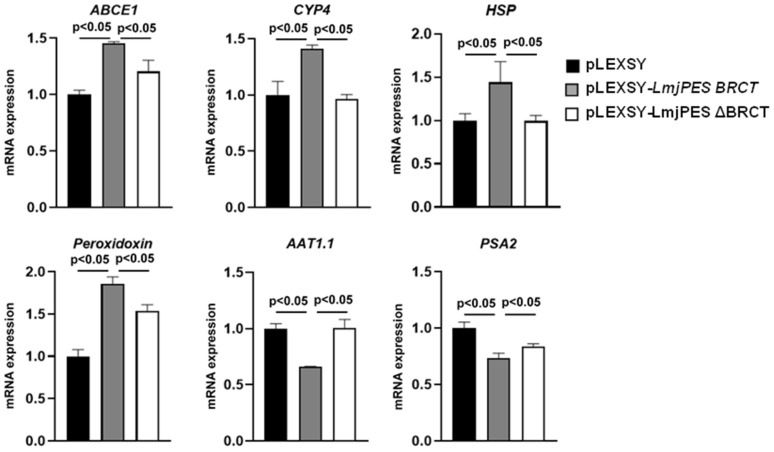
Gene expression quantification. mRNA levels of *ABCE1*, *CYP4*, *HSP*, *peroxidoxin*, *AAT1*.*1,* and *PSA2* genes in control (pLEXSY) parasites (*L*. *major*–MC*#4*, *#5,* and *#6* control cell lines*)* and in *L*. *major* parasites overexpressing *BRCT* domain from *LmjPES (L*. *major*–*LmjPES BRCT#54*, *#55,* and *#56* cell lines) or expressing a defective *LmjPES* lacking BRCT (*LmjPES ∆BRCT)* (*L*. *major*–*LmjPES ∆BRCT #1*, *#2,* and *#3* cell lines). Data are represented as means of triplicates of each cell line (±SD). *p*-values < 0.05 were considered statistically significant.

Afterwards, six genes belonging to the significantly enriched categories from Figure 2B (four up-regulated and two down-regulated in *LmjPES BRCT*-overexpressing parasites) were selected to further analyze the role played by this BRCT domain in parasite gene expression. Moreover, the mRNA levels of these genes were studied by real time-PCR in parasites with an overexpressed *BRCT* domain from *LmjPES* and in parasites expressing a mutated *LmjPES* lacking the coding sequence of *BRCT (LmjPES* Δ*BRCT)*, compared to parasites transfected with the DNA expression cassette containing only the hygromycin resistance sequence. As observed in the RNAseq, the four up-regulated genes (LMJF_21_0710: *ABCE1*, LMJF_33_1630: *CYP4*, LMJF_33_2390: *HSP,* and LMJF_23_0040: *peroxidoxin*) and the two down-regulated genes (LMJF_31_0320: *AAT1*.*1* and LMJF_12_0940: *PSA2*) were validated as expected (Figure 3). In addition, *ABCE1*, *CYP4*, *HSP,* and *peroxidoxin* mRNA expression levels decreased significantly in the parasites expressing a mutated *LmjPES* lacking *BRCT*, in comparison to *LmjPES BRCT-*overexpressing parasites (Figure 3). Similarly, the mRNA levels of *AAT1*.*1* and *PSA2* significantly increased in parasites expressing *LmjPES ∆BRCT* with respect to *LmjPES BRCT-*overexpressing parasites (Figure 3). Altogether, these data reinforced the role of the BRCT domain from LmjPES in the biology of the parasites.

### 3.3. BRCT Domain from LmjPES May Be Involved in L. major Pathogenesis

To better understand the implication of the BRCT domain from LmjPES on leishmaniasis outcomes, we subcutaneously inoculated mice with PBS or parasites harboring a DNA expression cassette containing the hygromycin resistance sequence and the *LmjPES BRCT* insert (or without such an insert for the control) by serial inoculations as described in Section 2. Materials and Methods. Animals were monitored weekly, and footpad swellings were measured with a digital caliper.

**Figure 4:**
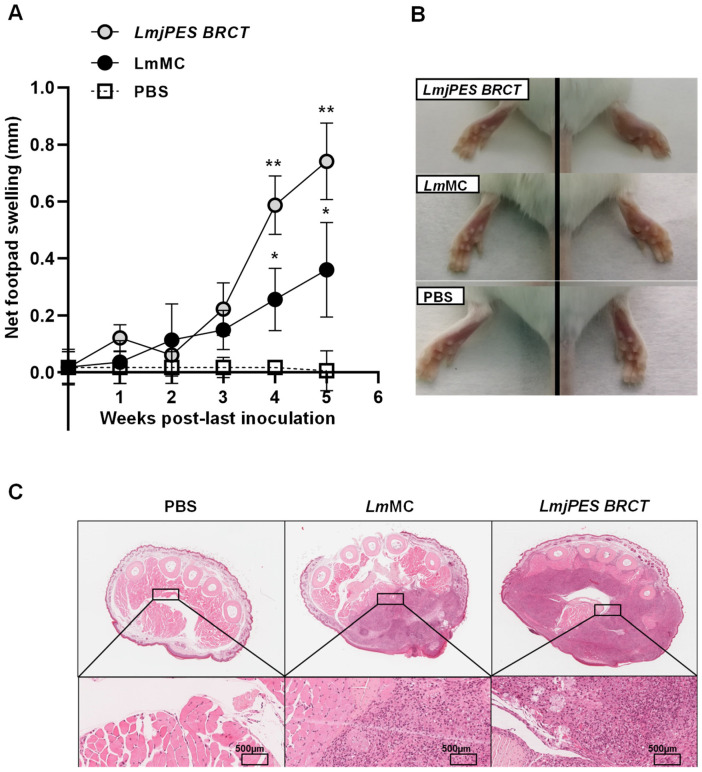
Net footpad swelling and hematoxylin–eosin staining of BALB/c mice infected with *LmjPES BRCT*-overexpressing parasites or control parasites. (**A**) The net footpad swelling corresponded to the difference between the infected (right) and non-infected (left) footpad, measured weekly until 35 days after the last parasite inoculation. Data are represented as means (±SD) of different animals in each group. ** *p* < 0.01 with respect to *L*. *major*—control cell line (LmMC, transfected with pLEXSY without insert). * *p* < 0.05 with respect to uninfected animals (inoculated with PBS). (**B**) Representative pictures of mouse footpad inoculated (right) and not inoculated (left) with *L*. *major*—LmjPES BRCT (overexpressing *LmjPES BRCT*), *L*. *major*—control (LmMC, transfected with pLEXSY without insert), and uninfected animals (inoculated with PBS). (**C**) Hematoxylin–eosin staining in footpad sections from mice infected with *L*. *major* overexpressing *LmjPES BRCT* or *L*. *major* control (LmMC) or non-infected mice inoculated with PBS.

The first reports of swelling were detected three weeks after the last inoculation. The inflammation generated after the infection by the *L*. *major*–LmjPES BRCT cell line (exhibiting an overexpression of *BRCT* from *LmjPES*) was significantly greater than the inflammation induced by *L*. *major*—MC cells (control cell lines; parasites transfected with pLEXSY without the insert) after weeks 4 and 5. The weekly monitorization of in vivo infections is presented in Figure 4A,B.

In agreement with these data, hematoxylin–eosin staining also showed a higher infiltrate area in the footpad inoculated with *LmjPES BRCT*-overexpressing parasites compared to the footpad infected with control parasites (LmMC, *L*. *major* transfected with pLEXSY without the insert) (Figure 4C).

## 4. Discussion

The aim of this paper was to study the potential role of the BRCT domain from LmjPES in *Leishmania*‘s biology. Integrative expression vectors were then designed and constructed to overcome two limitations of episomal constructs. Firstly, transgenic cells may lose the plasmid once there is no antibiotic pressure, for example, during a medium- or long-term murine infection [40]. Secondly, the expression of the transgene can be highly heterogenous within the population of engineered parasites, due to copy number variations [40]. Therefore, the transfection of DNA cassettes into the genome parasite has been proposed as an alternative to overcome these limitations [41].

After obtaining *Leishmania* constitutively overexpressing the *BRCT* domain from *LmjPES* (*L*. *major*–*LmjPES BRCT#54*, *#55,* and *#56* cell lines), RNAseq was performed to assess if this domain can alter the *L*. *major* gene expression levels. We found 152 genes differentially expressed. According to the GO and KEGG enrichment results, genes involved in metabolic processes were mostly affected, suggesting the role of the *BRCT* domain from *LmjPES* in those pathways. It has been described that metacyclogenesis mechanisms are associated with metabolic pathways to maintain and regulate changes in cellular morphology [38]. In addition, energy metabolism is required for the movement and infectivity of metacyclic promastigotes [38]. Through our results, we observed that *peroxidoxin* was one of the deregulated genes included in the metabolic pathway category. Peroxidoxin contributes in cell resistance to free radicals and has been described as a virulence factor [42,43]. Therefore, the up-regulation of this gene in our study may support the participation of the *BRCT* domain from *LmjPES* in the virulence of the parasite. On the other hand, RNAseq data showed a deregulation of genes clustered in protein folding, such as *HSP* and *CYP4*. Unfolded proteins can be generated under cellular stress conditions [44,45]. The increased levels of *HSP* and *CYP4* found in the overexpressed *LmjPES BRCT* domain in *Leishmania* suggested that this domain might be involved in replication processes, elevated levels of reactive oxygen species (ROS), and cell stress-related situations.

In addition, the expression levels of genes encoding some transporters such as *ABCE1* and *AAT1*.*1* were altered. These proteins have key physiological functions and play essential roles in response to drugs [46,47,48], indicating a putative effect of the BRCT domain from LmjPES on *Leishmania* viability. Another category affected was the response to stimuli, including the *PSA2* gene, a *Leishmania* antigen, which can induce Th1-mediated protection against murine leishmaniasis [49]. The down-regulation of *PSA2* might lead to disease development.

Interestingly, the depletion of the *BRCT* domain from *LmjPES* (*L*. *major*–*LmjPES ∆BRCT #1*, *#2,* and *#3* cell lines) recovered the expression of these genes to normal levels, corroborating the role of this domain in the pathophysiology of leishmaniasis. After observing the RNAseq results, we were prompted to evaluate the implications of the LmjPES BRCT domain in *L*. *major* in vivo infectivity and virulence. The inflammation caused by *L*. *major*–*LmjPES BRCT*-overexpressing cell lines became significantly larger than those of the control by weeks 4 and 5. The correlation between the in vivo lesion size and genetic overexpression have been extensively reported in *L*. *major* for genes implicated in both disease progression [50] and a reduction in virulence [37,51,52]. Interestingly, footpad swelling is thought to be linked with both local inflammation and parasite replication [37]. Furthermore, in mammalian PES, it has been described that point mutations within the BRCT domain of such a protein had a negative impact on rRNA processing and protein stability and prevented mammalian PES1 from being incorporated into the PeBoW complex [22]. Moreover, it is known that the addition of an extra PES allele lacking the whole BRCT domain can induce negative effects on cell fitness [15]. Thus, this underlines the importance of this domain for the adequate function of protein.

## 5. Conclusions

Taken together, and considering the previous data showing the role of LmjPES in *L*. *major* in vitro infectivity [14], our results highly support the link between the LmjPES BRCT domain and *Leishmania* infectivity and pathogenesis. This work highlighted the LmjPES BRCT domain as a virulence factor, playing a key role during the *L*. *major* infection process and pathogenesis, while significant inflammation in mice infected with parasites overexpressing this domain was observed. Finally, further experiments need to be conducted to analyze this domain in depth as a potentially relevant therapeutic target.

## Figures and Tables

**Table 1:** Oligos used for plasmids’ construction.

Oligo Name	Oligo Sequence (5′-3′)
*BRCTF1*	ACCAGATCTGCCATGG*ACACCATG***CGCGGGCTAACCTTCTTCATATCG**
*BRCTR2*	TGGTGATGGTGGTGGGTACC**GCGGTAGCCCGTCACCGG**
*PES∆BRCTF1*	CCATGG *GAA**ATG*** **GTCCATAAGAAGCAGGCA**
*PES∆BRCTR2*	**GCGGAACAGCTCGCGCA**
*PES∆BRCTF3*	TGCGCGAGCTGTTCCGC**AACGCGCGGCTGGTG**
*PES∆BRCTR4*	GGTACC **CTGCACCCACTTGGGCAGTTT**

In bold: *BRCTF1*, sequence from the start of *BRCT* domain; *BRCTR2*, sequence from the end of *BRCT* domain; *PES∆BRCTF1*, the beginning of *LmjPES* coding sequence; *PES∆BRCTR2*, sequence until the first codon of *BRCT* domain; *PES∆BRCTF3,* the end sequence of *BRCT* domain; *PES∆BRCTR4*, the end of *LmjPES* coding sequence. Underlined: Endonuclease restriction site. Italics: Start codon plus Kozak sequence.

**Table 2:** Primers used for quantitative real time-PCR.

Target Gene	Sense Primer (5′-3′)	Antisense Primer (5′-3′)	Amplicon Length (pb)
*AAT1*.*1*	GCAGGTGATTATGCCGTATG	GCACAAAGGAGTAAATCGCC	170
*HSP*	CCTTTAAAGTGACGGAGTGC	TCGACAGTGTTTACCTTGCC	235
*ABCE1*	TTCGTATCATCAACCTCCCC	CCCAGGCTCATTCATGTATC	209
*CYP4*	TTCACTGAAAGTGTCCCTCC	TTGAAGAGCTCCATCTCGAC	162
*PSA2*	GCACTCGATGACATCTTTGG	TTAAGAGAGACGGAAGCCAG	254
*Peroxidoxin*	ACATGAACGACTACAAGGGC	GATTCTTCGATCAGCACACC	279
*GAPDH*	CATCAAGTGCGTGAAGGCGC	CGTCGGCGAGTACTCGTGCTG	216
*LmjPES BRCT*	TCTTCATATCGCGTGAGGTG	CATGCTTTTTCATCCCTGGC	147

## Data Availability

RNA sequencing data are publicly available in the GEO database with the accession number GSE234048.

## Supplementary Materials

The following supporting information can be downloaded at https://www.mdpi.com/article/10.3390/biom15081191/s1: Table S1: Differential gene expression analysis.

## Abbreviations

The following abbreviations were used in this manuscript:

BRCT	Breast cancer associated 1 C-terminal domain,
CL	Cutaneous Leishmaniasis
PES1	Pescadillo ribosomal biogenesis factor 1
VL	Visceral Leishmaniasis
WHO	World Health Organization
