# Supplementary material for: The Role of BRCT Domain from LmjPES in Leishmania major Pathogenesis
Source: Biomolecules. 2025 Aug 19;15(8):1191. doi: 10.3390/biom15081191 (PMC12384356; doi:10.3390/biom15081191)
Supplement: Supplementary file 1 [file biomolecules-15-01191-s001.zip › biomolecules-3780182-supplementary.pdf]

Table S1. Differential gene expression analysis. Each row represents a gene, including its identifier (gene_id), type (gene_type), name (gene_name), and functional description (gene_desc). Additional columns include the annotation source (gene_source), chromosome (chr), start and end positions (start, end), and strand orientation (strand). The statistical outputs include logFC (log fold-change), AveExpr (average expression), t (t-statistic), P.Value (raw p-value), adj.P.Val (FDR-adjusted p-value), and B (log-odds of differential expression). Red-highlighted rows indicate upregulated genes (positive logFC), while green-highlighted rows represent downregulated genes (negative logFC).

gene_id	gene_type	gene_name	gene_desc	gene_source	chr	start	end	strand	logFC	AveExpr	t	P.Value	adj.P.Val	B
ENSRNAG00049765837	rRNA	LSU_rRNA_eukarya	NA	Rfam	27	1004838	1009202	-	0.4310343	3.2054888	4.39833943	0.00012556	0.010308195	1.043499019
LMJF_01_0690	protein_coding	NA	hypothetical protein	ena	1	202894	203586	+	-0.429841	2.2036081	-3.99643285	0.00038337	0.020711872	0.087602645
LMJF_02_0010	protein_coding	SCG3	phosphoglycan beta 1,3 galactosyltransferase 3	ena	2	4431	6875	-	-0.423635	3.6839892	-5.30581716	9.70E-06	0.001504303	3.443676908
LMJF_02_0160	protein_coding	SCGR6	phosphoglycan beta 1,3 galactosyltransferase	ena	2	77219	79672	-	0.6159343	3.1515338	6.90006543	1.14E-07	3.97E-05	7.698695451
LMJF_02_0450	protein_coding	NA	putative voltage-dependent anion-selective channel	ena	2	200383	201216	-	0.4490683	3.9536677	5.80116636	2.40E-06	0.000519774	4.785171102
LMJF_02_0660	protein_coding	NA	hypothetical protein	ena	2	330066	332858	+	0.5972014	1.5175712	4.28815087	0.00017082	0.011983335	0.846948801
LMJF_03_0200	protein_coding	NA	putative delta-1-pyrroline-5-carboxylate dehydrogenase	ena	3	48406	50088	+	0.3404892	3.8242054	4.49331729	9.62E-05	0.008138429	1.244239685
LMJF_03_0310	protein_coding	NA	hypothetical protein	ena	3	82957	89220	+	-0.461534	2.6290015	-4.64046185	6.36E-05	0.006053754	1.723032338
LMJF_03_0410	protein_coding	NA	putative MFS transporter	ena	3	131769	133820	+	-0.636719	2.8725735	-7.10659678	6.49E-08	2.36E-05	8.225482896
LMJF_04_0310	protein_coding	NA	putative beta-fructofuranosidase	ena	4	103123	105045	+	-0.449017	6.0582246	-7.21362174	4.86E-08	1.85E-05	8.514845103
LMJF_04_0320	protein_coding	NA	putative beta-fructofuranosidase	ena	4	106566	108026	+	-0.461418	2.8551089	-5.08074149	1.83E-05	0.002366465	2.881296328
LMJF_04_0330	protein_coding	NA	putative mitochondrial exoribonuclease DSS-1	ena	4	109167	111740	+	-0.34457	3.4208356	-4.30523559	0.00016287	0.01180224	0.761619809
LMJF_04_0810	protein_coding	NA	hypothetical protein	ena	4	317131	319146	-	1.1036148	3.3646221	12.417	2.22E-13	1.26E-09	19.7496367
LMJF_05_0760	protein_coding	NA	kinesin-like protein	ena	5	280459	284223	+	-0.352773	3.2341727	-4.26731594	0.00018103	0.012128325	0.677486981
LMJF_06_0050	protein_coding	PUF5	putative pumillio protein 5	ena	6	21431	22843	-	-0.339701	3.329755	-4.17610269	0.0002333	0.014689409	0.428274263
LMJF_06_0310	protein_coding	NA	putative folate/biopterin transporter	ena	6	103880	105910	-	-0.493564	3.3071693	-5.97610379	1.47E-06	0.000361087	5.270403227
LMJF_07_0090	protein_coding	NA	putative methionine synthase	ena	7	28603	32361	+	-0.356436	3.125432	-4.10116475	0.00028715	0.016933875	0.2516045
LMJF_07_0860	protein_coding	NA	hypothetical protein	ena	7	436928	441199	+	-0.573624	4.7195435	-8.10264943	4.66E-09	3.54E-06	10.80257782
LMJF_07_0950	protein_coding	NA	hypothetical protein	ena	7	500162	505981	+	-0.305593	4.1187193	-4.18008697	0.00023074	0.014637891	0.381095588
LMJF_08_0690	protein_coding	NA	amastin-like protein	ena	8	298369	298983	+	-0.39968	4.1640488	-4.85857929	3.44E-05	0.003838721	2.202723453
LMJF_09_1010	protein_coding	NA	hypothetical protein	ena	9	408693	410540	+	0.4197134	4.0907518	5.58112521	4.45E-06	0.000832994	4.182615621
LMJF_10_0465	protein_coding	GP63-2	GP63, leishmanolysin	ena	10	216396	218204	+	-0.508953	2.5463294	-5.05265991	1.99E-05	0.002410072	2.822360375
LMJF_11_0630	protein_coding	NA	putative aminopeptidase	ena	11	236568	238184	+	0.3300084	4.1878491	4.07870594	0.00030555	0.017384215	0.117291136
LMJF_12_0280	protein_coding	NA	putative ornithine decarboxylase	ena	12	104356	106479	+	0.3756514	3.3297852	4.50094856	9.42E-05	0.00804725	1.302816681
LMJF_12_0500	protein_coding	NA	hypothetical protein	ena	12	258497	261607	-	0.5850318	3.4925881	7.2442702	4.48E-08	1.85E-05	8.60144181
LMJF_12_0530	protein_coding	PGI	glucose-6-phosphate isomerase	ena	12	293730	295547	+	0.2859615	4.5674994	4.1019329	0.00028654	0.016933875	0.151705745
LMJF_12_0630	protein_coding	ALAT	alanine aminotransferase	ena	12	343177	344670	+	0.5103807	2.8351438	5.36531326	8.20E-06	0.001295258	3.652388648
LMJF_12_0760	protein_coding	NA	putative surface antigen protein 2	ena	12	471517	473280	+	0.5727698	3.8305223	7.54913282	1.98E-08	9.23E-06	9.389450816
LMJF_12_0940	protein_coding	NA	promastigote surface antigen protein 2 PSA2	ena	12	442787	443944	+	-0.311614	4.2301671	-4.29420877	0.00016796	0.011983335	0.677110796
LMJF_12_1070	protein_coding	NA	putative surface antigen protein 2	ena	12	464061	466175	+	-0.327673	4.2290942	-4.47312244	0.00010182	0.008442321	1.15643815
LMJF_13_0090	protein_coding	NA	metallo-peptidase, Clan MA(E), family 32	ena	13	23388	24899	-	-0.472975	2.9421335	-5.38331262	7.79E-06	0.001254642	3.689399612
LMJF_13_1690	protein_coding	NA	hypothetical protein	ena	13	645551	647374	+	-1.235884	2.3133823	-12.2670581	3.02E-13	1.26E-09	19.02412901
LMJF_14_0380	protein_coding	NA	conserved hypothetical protein	ena	14	123258	125081	+	0.4511329	3.2946424	4.76256067	4.51E-05	0.004643318	2.008362226
LMJF_14_0460	protein_coding	NA	cystathionine beta-lyase-like protein	ena	14	152236	153615	+	-0.806907	1.6789547	-6.3638826	4.97E-07	0.000140691	6.134092791
LMJF_14_0670	protein_coding	ELO2	beta-ketoacyl-CoA synthase	ena	14	244332	245216	-	0.3407001	4.3895268	4.38033866	0.00013205	0.010632192	0.905682671
LMJF_14_0810	protein_coding	NA	putative kinesin	ena	14	321571	325518	-	-0.321296	3.5742769	-4.08844929	0.00029743	0.017296355	0.17746409
LMJF_15_1380	protein_coding	NA	putative nucleolar RNA binding protein	ena	15	571791	573599	-	0.3878887	3.0280525	4.08179887	0.00030295	0.017384215	0.230945776
LMJF_16_0950	protein_coding	NA	sucrose-phosphate synthase-like protein	ena	16	346095	347489	+	0.4988532	3.0687429	5.62958894	3.89E-06	0.00075668	4.352632983
LMJF_16_1020	protein_coding	NA	hypothetical protein	ena	16	389044	391614	+	0.4739969	2.2709246	4.26729852	0.00018104	0.012128325	0.788446697
LMJF_16_1310	protein_coding	NA	putative cytochrome c	ena	16	532864	533205	-	0.5597399	2.0918871	4.57175565	7.72E-05	0.007262507	1.578050929
LMJF_17_0250	protein_coding	CYSB	cystathionine beta-synthase	ena	17	133086	134165	-	0.5420125	2.668917	5.55025088	4.86E-06	0.000847982	4.144035304
LMJF_17_0870	protein_coding	META2	META domain containing protein	ena	17	415950	417284	+	0.3581783	3.1479925	4.07670328	0.00030724	0.017384215	0.201413175
LMJF_17_0890	protein_coding	META1	hypothetical protein	ena	17	423158	423496	+	0.7501484	2.0456483	5.84650445	2.11E-06	0.000477404	4.847710619
LMJF_18_0510	protein_coding	NA	putative aconitase	ena	18	209236	211926	-	0.2997136	4.6549874	4.34551773	0.00014555	0.011181637	0.794452099
LMJF_18_0670	protein_coding	NA	putative citrate synthase	ena	18	293991	295355	+	0.4013301	2.6333532	4.08947159	0.00029659	0.017296355	0.298343203
LMJF_18_0680	protein_coding	NA	putative citrate synthase	ena	18	287997	289409	+	0.5289285	3.9163601	6.83417456	1.36E-07	4.40E-05	7.545732751
LMJF_18_1370	protein_coding	NA	putative heat shock protein	ena	18	608452	610923	+	0.3277599	4.5136017	4.54637966	8.29E-05	0.007628087	1.344371142
LMJF_19_0040	protein_coding	NA	histone H2B	ena	19	6427	6750	-	-0.319149	4.6954112	-4.10445857	0.00028455	0.016933875	0.141056387
LMJF_19_0200	protein_coding	ANC1	putative ADP/ATP translocase 1	ena	19	51499	52452	-	0.589927	2.2544427	5.02816327	2.13E-05	0.002546152	2.762861498
LMJF_19_0210	protein_coding	ANC2	putative ADP/ATP translocase 1	ena	19	56935	57888	-	0.3451523	5.6129144	5.10391958	1.72E-05	0.002358031	2.80311117
LMJF_19_0270	protein_coding	NA	hypothetical protein	ena	19	91746	94631	+	-0.639854	4.5259464	-9.04512895	4.32E-10	9.04E-07	13.08446083
LMJF_19_0600	protein_coding	NA	hypothetical protein	ena	19	257800	260091	+	-0.645133	2.519299	-6.45485806	3.86E-07	0.000115501	6.519120815
LMJF_19_0630	protein_coding	NA	putative histone H3 variant	ena	19	270430	270864	+	-0.370334	2.9934673	-4.27270322	0.00017834	0.012128325	0.716015116
LMJF_19_1560	protein_coding	NA	inosine-5'-monophosphate dehydrogenase	ena	19	673010	674554	+	0.4006529	3.3128688	4.84473664	3.58E-05	0.00393931	2.226509698
LMJF_20_0700	protein_coding	NA	hypothetical protein	ena	20	268471	271653	+	-0.385298	4.0022212	-4.75481643	4.61E-05	0.004649672	1.930227302
LMJF_20_1420	protein_coding	NA	hypothetical protein	ena	20	644883	648560	+	-0.397046	2.7794214	-4.35883655	0.00014023	0.010872856	0.965800478
LMJF_21_0430	protein_coding	NA	hypothetical protein	ena	21	138525	139718	+	0.5442801	2.2602218	4.75959649	4.55E-05	0.004643318	2.063284044
LMJF_21_0440	protein_coding	NA	ubiquitin-conjugating enzyme-like protein	ena	21	141192	142004	+	-0.389784	2.6325923	-4.20108043	0.00021767	0.014129905	0.569140538
LMJF_21_0710	protein_coding	ABCE1	putative ATP-binding cassette protein subfamily E,member 1	ena	21	219213	221192	-	0.5611847	2.9529681	5.57822087	4.49E-06	0.000832994	4.218206685
LMJF_21_0740	protein_coding	NA	putative ATPase subunit 9	ena	21	234023	234343	+	0.5130681	2.8763049	5.06774091	1.90E-05	0.002366465	2.857097165
LMJF_22_0180	protein_coding	NA	hypothetical protein	ena	22	79177	79959	-	0.5177084	3.9262018	6.35878304	5.04E-07	0.000140691	6.286844167
LMJF_22_0230	protein_coding	AAT22	putative amino acid permease	ena	22	101894	103351	-	0.3981076	5.0988747	5.66662226	3.50E-06	0.00071489	4.37386141
LMJF_23_0010	protein_coding	NA	hypothetical protein	ena	23	2243	4084	+	0.5934619	2.4249718	5.15640172	1.48E-05	0.002101446	3.101800998
LMJF_23_0040	protein_coding	NA	peroxidoxin	ena	23	11076	11756	+	0.349665	4.4466916	4.79870351	4.07E-05	0.004371243	2.032442634
LMJF_23_0050	protein_coding	CYP11	putative cyclophilin 11	ena	23	12570	13457	+	0.6719687	1.5248135	4.52252496	8.86E-05	0.007929366	1.42685812
LMJF_23_0200	protein_coding	NA	putative endoribonuclease L-PSP (pb5)	ena	23	61657	62148	+	0.4132243	3.5129157	5.11138514	1.68E-05	0.00234721	2.933362636
LMJF_23_0360	protein_coding	NA	putative NADP-dependent alcohol dehydrogenase	ena	23	131148	132206	+	-0.68198	3.0911195	-8.18026135	3.81E-09	3.54E-06	10.91818445
LMJF_23_0450	protein_coding	NA	permease-like protein	ena	23	166701	170015	+	-0.424897	4.2124518	-5.88753781	1.88E-06	0.000437236	5.005149906
LMJF_24_0360	protein_coding	NA	putative UDP-galactose transporter	ena	24	110765	112567	+	0.4927015	2.6064029	4.81538996	3.88E-05	0.004224199	2.199869676
LMJF_24_1280	protein_coding	NA	amastin-like surface protein-like protein	ena	24	454236	454796	+	-0.399862	4.3579692	-5.63135416	3.87E-06	0.00075668	4.302447612
LMJF_24_1570	protein_coding	NA	conserved hypothetical protein	ena	24	587425	589503	+	-0.306421	4.56221	-4.30387632	0.00016349	0.01180224	0.681072306
LMJF_24_1880	protein_coding	CYC11	putative cyclin 11	ena	24	695194	697992	-	-0.363168	2.7245722	-4.02071161	0.00035858	0.019754782	0.090771632
LMJF_25_1120	protein_coding	ALDH2	aldehyde dehydrogenase, mitochondrial precursor	ena	25	452597	454096	-	-0.393711	3.2754963	-4.72654734	4.99E-05	0.004860021	1.899258987
LMJF_25_1770	protein_coding	EnCR	polyprenol reductase	ena	25	710124	711044	-	0.4548661	3.3724466	5.568051	4.62E-06	0.000832994	4.1760824
LMJF_26_2110	protein_coding	NA	putative protein kinase	ena	26	838659	843977	+	-0.309532	4.1352654	-4.25417121	0.00018778	0.012381886	0.576706734
LMJF_26_2590	protein_coding	NA	conserved hypothetical protein	ena	26	1043167	1045635	+	0.476778	2.4058986	4.38457786	0.00013049	0.010608863	1.082602015
LMJF_27_0510	protein_coding	NA	putative calpain-like cysteine peptidase	ena	27	194587	210663	+	0.2572567	6.6523485	4.14213348	0.00025636	0.015669511	0.159921595
LMJF_27_1260	protein_coding	NA	putative T-complex protein 1, beta subunit	ena	27	527916	529505	-	0.4017336	3.4487189	4.55687979	8.05E-05	0.007488548	1.443030019
LMJF_27_1870	protein_coding	TRYS	putative trypanothione synthetase	ena	27	806164	808122	-	0.3545099	4.1655778	4.50340633	9.35E-05	0.00804725	1.250334468
LMJF_27_2400	protein_coding	NA	putative heat shock protein DNAJ	ena	27	1071304	1072494	+	0.4863378	4.9313853	6.81450991	1.44E-07	4.45E-05	7.482476196
LMJF_27_rRNA_13	rRNA	NA	28S ribosomal RNA (LSU-alpha)	ena	27	992934	994713	+	0.5705417	3.2456417	4.31949634	0.00015652	0.01180224	0.835871681
LMJF_27_rRNA_20	rRNA	NA	28S ribosomal RNA (LSU-gamma, M1)	ena	27	1006705	1006917	+	0.950553	0.916486	4.1840465	0.00022822	0.014588367	0.502188021
LMJF_27_rRNA_24	rRNA	NA	28S ribosomal RNA (LSU-gamma, M1)	ena	27	1057514	1057726	+	0.4744979	7.0079176	4.16939238	0.00023769	0.014780227	0.23953803
LMJF_27_rRNA_32	rRNA	NA	28S ribosomal RNA (LSU-delta, M2)	ena	27	1008798	1008980	+	0.5892381	6.3728967	5.20771841	1.28E-05	0.001881126	3.068717409
LMJF_27_rRNA_42	rRNA	NA	28S ribosomal RNA (LSU-zeta, M6)	ena	27	1060334	1060406	+	0.3929777	4.8907165	5.08939391	1.79E-05	0.002366465	2.801654921
LMJF_28_0090	protein_coding	NA	adenylate cyclase-like protein	ena	28	27737	29587	+	0.4840086	2.3602632	4.28033139	0.00017458	0.011983335	0.816373396
LMJF_28_0140	protein_coding	NA	putative pantothenate kinase subunit	ena	28	36083	40468	+	0.5594625	4.9067613	8.12408511	4.40E-09	3.54E-06	10.85550186
LMJF_28_0230	protein_coding	NA	hypothetical protein	ena	28	71386	72369	+	-0.509717	1.75413	-4.31845331	0.00015697	0.01180224	0.93180359
LMJF_28_0790	protein_coding	NA	putative dual-specificity protein phosphatase	ena	28	284515	286404	+	0.679076	1.1148057	4.20423724	0.00021577	0.014115919	0.603309615
LMJF_28_0980	protein_coding	P27	putative P27 protein	ena	28	353036	353758	+	0.6649914	1.7313381	5.01198513	2.23E-05	0.002627864	2.679456933
LMJF_28_1490	protein_coding	NA	hypothetical protein	ena	28	559265	559681	+	0.6234825	3.0445422	6.83256783	1.37E-07	4.40E-05	7.51693105
LMJF_28_2310	protein_coding	NA	putative eukaryotic translation initiation factor	ena	28	896720	897937	+	0.352748	3.5257751	4.28313029	0.00017323	0.011983335	0.70603682
LMJF_28_2430	protein_coding	NA	putative vacuolar ATP synthase subunit b	ena	28	943761	945248	+	0.3588155	3.3404249	4.35915856	0.0001401	0.010872856	0.924093543
LMJF_29_0620	protein_coding	ABCA10	putative ATP-binding cassette protein subfamily A,member 10	ena	29	230353	235953	-	0.3317562	5.7168393	5.21888563	1.24E-05	0.001855148	3.114582603
LMJF_29_0868	protein_coding	NA	hypothetical protein	ena	29	327859	328365	-	0.5403135	1.651664	4.15608003	0.00024663	0.015186027	0.525049124
LMJF_29_1340	protein_coding	NA	putative RNA-binding protein	ena	29	542136	544055	+	-0.58101	3.6412018	-7.21985665	4.78E-08	1.85E-05	8.548504275
LMJF_29_1500	protein_coding	NA	hypothetical protein	ena	29	686960	688978	-	0.7444965	3.1170942	8.01959797	5.77E-09	3.56E-06	10.48677875
LMJF_29_1510	protein_coding	NA	hypothetical protein	ena	29	693379	695397	-	0.6519293	4.2854263	8.11463525	4.51E-09	3.54E-06	10.81840892
LMJF_30_1850	protein_coding	NA	putative phosphatidylinositol kinase	ena	30	688915	693039	+	-0.45726	3.1888142	-5.50774425	5.48E-06	0.000936638	4.0134293
LMJF_30_1940	protein_coding	NA	succinyl-coa:3-ketoacid-coenzyme a transferase-like protein	ena	30	719484	720983	+	0.3626366	3.8053125	4.52104633	8.90E-05	0.007929366	1.320167656
LMJF_30_2550	protein_coding	NA	putative heat shock 70-related protein 1,mitochondrial precursor	ena	30	979560	981518	-	0.2600246	6.1798543	4.11109843	0.00027937	0.016933875	0.082748229
LMJF_30_2980	protein_coding	NA	glyceraldehyde 3-phosphate dehydrogenase,glycosomal	ena	30	1122342	1123427	-	0.2809308	5.458152	4.16850394	0.00023828	0.014780227	0.267727987
LMJF_30_3040	protein_coding	NA	eukaryotic translation initiation factor 3 subunit 7-like protein	ena	30	1140481	1142076	-	0.4229843	3.9334125	5.42535055	6.92E-06	0.001135869	3.765197668
LMJF_30_3520	protein_coding	METK2	S-adenosylmethionine synthetase	ena	30	1336012	1337190	+	-0.846211	5.1657694	-11.7237288	9.39E-13	2.62E-09	18.95909587
LMJF_31_0010	protein_coding	NA	5-methyltetrahydropteroyltriglutamate-homocysteine S-methyltransferase	ena	31	3246	5558	-	-0.633405	3.5060736	-7.90773744	7.72E-09	4.13E-06	10.28533537
LMJF_31_0310	protein_coding	NA	hypothetical protein	ena	31	84525	88190	-	0.3359781	3.182896	4.03295893	0.00034667	0.019225525	0.083004877
LMJF_31_0320	protein_coding	AAT1.1	putative amino acid transporter	ena	31	90450	92057	-	-0.591752	4.7427835	-8.63940107	1.18E-09	1.65E-06	12.12245743
LMJF_31_0330	protein_coding	AAT1.2	putative amino acid transporter aATP11	ena	31	95194	96696	-	-0.355451	3.3925492	-4.29125887	0.00016935	0.011983335	0.726736936
LMJF_31_0350	protein_coding	AAT1.4	putative amino acid transporter aATP11	ena	31	107840	109375	-	-0.567105	4.6111438	-7.89927593	7.90E-09	4.13E-06	10.29152134
LMJF_31_0570	protein_coding	AAT25.1	putative amino acid transporter aATP11	ena	31	201590	203008	-	0.75239	1.750911	5.78854816	2.48E-06	0.000519774	4.635106575
LMJF_31_0580	protein_coding	AAT25.2	putative amino acid transporter aATP11	ena	31	207417	209042	-	-0.330541	4.7443797	-4.77667698	4.33E-05	0.004535404	1.947123604
LMJF_31_0720	protein_coding	NA	hypothetical protein	ena	31	258241	261180	-	-0.373559	2.935845	-4.31326204	0.00015926	0.01180224	0.828885427
LMJF_31_0900	protein_coding	NA	hypothetical protein	ena	31	332843	333082	-	-0.617564	3.8403066	-8.23083051	3.35E-09	3.54E-06	11.09767416
LMJF_31_1030	protein_coding	NA	hypothetical protein	ena	31	399111	401819	-	-0.349555	4.4284517	-4.78688692	4.21E-05	0.004462329	1.9932723
LMJF_31_1070	protein_coding	NA	biotin/lipoate protein ligase-like protein	ena	31	421744	422535	-	0.5114542	2.4024991	4.73772674	4.84E-05	0.004764553	2.0034069
LMJF_31_1220	protein_coding	NA	putative vacuolar-type proton translocating pyrophosphatase 1	ena	31	487881	490289	-	0.4202884	4.7527127	5.939774	1.62E-06	0.000388392	5.133994148
LMJF_31_1840	protein_coding	NA	hypothetical protein	ena	31	877913	881989	-	-0.325486	3.3929463	-4.10167838	0.00028674	0.016933875	0.226943374
LMJF_31_1920	protein_coding	NA	hypothetical protein	ena	31	940976	944908	-	-0.307667	4.2776214	-4.05443685	0.00032673	0.018362703	0.038313269
LMJF_31_2410	protein_coding	NA	hypothetical protein	ena	31	1201131	1202141	-	0.5149467	2.9897865	5.27416871	1.06E-05	0.001615319	3.40376069
LMJF_32_1920	protein_coding	NA	hypothetical protein	ena	32	751077	752483	-	0.4591777	2.3624316	4.28333529	0.00017313	0.011983335	0.823439193
LMJF_32_2660	protein_coding	AAT16	putative amino acid transporter	ena	32	1049741	1051207	-	0.4637553	5.854405	7.32824756	3.57E-08	1.58E-05	8.819517554
LMJF_33_1140	protein_coding	NA	hypothetical protein	ena	33	528588	529886	+	0.6223758	4.9601413	8.91436223	5.96E-10	9.99E-07	12.78174364
LMJF_33_1630	protein_coding	CYP4	putative cyclophilin 4	ena	33	771363	772025	+	0.4804462	2.861125	4.72240593	5.05E-05	0.004860603	1.937661662
LMJF_33_2150	protein_coding	NA	hypothetical protein	ena	33	966924	969545	+	-0.413523	2.371043	-4.07955186	0.00030484	0.017384215	0.283284935
LMJF_33_2390	protein_coding	NA	putative heat shock protein	ena	33	1081986	1083890	+	0.6561129	3.277242	7.80450715	1.01E-08	4.98E-06	9.990736105
LMJF_34_0460	protein_coding	NA	hypothetical protein	ena	34	159488	166531	+	-0.409605	3.3326241	-4.99359368	2.35E-05	0.002729765	2.615509952
LMJF_34_0840	protein_coding	eEF1B	NA	ena	34	374108	374821	+	0.5159351	3.7046544	6.17012309	8.52E-07	0.000216223	5.786262459
LMJF_34_1410	protein_coding	NA	d-isomer specific 2-hydroxyacid dehydrogenase-like protein	ena	34	645653	646660	-	0.8735874	2.4835193	8.04051469	5.47E-09	3.56E-06	10.34940595
LMJF_34_2580	protein_coding	NA	conserved hypothetical protein	ena	34	1187377	1187994	+	0.419513	5.2146039	6.29644072	5.99E-07	0.000161899	6.085728798
LMJF_34_2700	protein_coding	NA	conserved hypothetical protein	ena	34	1232666	1233433	+	0.5038833	2.107588	4.36111053	0.00013934	0.010872856	1.036828762
LMJF_34_3400	protein_coding	NA	hypothetical protein	ena	34	1494552	1495529	+	-0.583742	1.9531726	-5.09406678	1.77E-05	0.002366465	2.931977339
LMJF_35_0640	protein_coding	NA	beta-fructofuranosidase-like protein	ena	35	302145	305417	+	-0.381447	4.5605876	-5.43063217	6.82E-06	0.001135869	3.745039005
LMJF_35_1300	protein_coding	NA	putative ubiquitin-conjugating enzyme e2	ena	35	572946	573392	+	0.5545235	3.6381184	6.19530359	7.94E-07	0.000207856	5.855162039
LMJF_35_1460	protein_coding	NA	hypothetical protein	ena	35	633311	634300	+	0.4266402	3.0693101	4.85986205	3.43E-05	0.003838721	2.28674762
LMJF_35_1510	protein_coding	NA	hypothetical protein	ena	35	655979	657730	-	-0.358147	4.0490887	-4.74028847	4.80E-05	0.004764553	1.888435091
LMJF_35_3100	protein_coding	NA	putative ATP-dependent RNA helicase	ena	35	1270926	1273697	-	0.2585022	6.1455914	4.19601853	0.00022075	0.014219804	0.310152235
LMJF_35_3680	protein_coding	NA	putative ribulose-phosphate 3-epimerase	ena	35	1489694	1490455	-	0.7601757	1.299858	5.07452694	1.87E-05	0.002366465	2.749157446
LMJF_35_3860	protein_coding	NA	putative T-complex protein 1, eta subunit	ena	35	1532610	1534322	-	0.3582748	3.6196344	4.48906114	9.74E-05	0.008153856	1.247106396
LMJF_35_4240	protein_coding	NA	hypothetical protein	ena	35	1705016	1705912	+	-0.500326	2.2496995	-4.91029581	2.97E-05	0.003407555	2.454889224
LMJF_35_4380	protein_coding	NA	hypothetical protein	ena	35	1762158	1763645	+	0.5931669	2.6326987	5.5640232	4.68E-06	0.000832994	4.179352953
LMJF_35_4470	protein_coding	NA	hypothetical protein	ena	35	1794331	1794930	+	0.296383	4.7315486	4.30868688	0.00016131	0.01180224	0.690723087
LMJF_36_0190	protein_coding	EF2-2	elongation factor 2	ena	36	58232	60769	-	0.3245354	6.7109854	5.07385445	1.87E-05	0.002366465	2.696347333
LMJF_36_1430	protein_coding	NA	putative translation elongation factor 1-beta	ena	36	526909	527544	-	0.3679394	3.7562614	4.50414354	9.33E-05	0.00804725	1.278345717
LMJF_36_2020	protein_coding	NA	chaperonin HSP60, mitochondrial precursor	ena	36	795457	797154	+	0.3695179	4.3555301	4.53718154	8.51E-05	0.00774273	1.329868604
LMJF_36_2590	protein_coding	NA	putative membrane-bound acid phosphatase 2	ena	36	1046527	1048242	-	0.462813	2.6281076	4.50924381	9.20E-05	0.00804725	1.392102714
LMJF_36_4230	protein_coding	NA	hypothetical protein	ena	36	1608348	1611719	+	0.4566821	3.7352978	5.79291227	2.45E-06	0.000519774	4.769969503
LMJF_36_5100	protein_coding	NA	hypothetical protein	ena	36	1968498	1971443	-	0.3056322	4.622198	4.30953373	0.00016093	0.01180224	0.700365457
LMJF_36_6060	protein_coding	NA	putative eukaryotic initiation factor 4a	ena	36	2331587	2333884	-	0.3158806	4.4154372	4.36057979	0.00013955	0.010872856	0.8506929
LMJF_36_6390	protein_coding	NA	putative methylenetetrahydrofolate reductase	ena	36	2480583	2481512	-	-1.124768	1.2734745	-8.00795684	5.95E-09	3.56E-06	9.785008819
LMJF_36_6760	protein_coding	NA	hypothetical protein	ena	36	2600792	2601568	-	0.3692019	3.203548	4.25560111	0.00018704	0.012381886	0.664537105
LMJF_36_6980	protein_coding	NA	putative eukaryotic translation initiation factor 3 subunit 8	ena	36	2670219	2672414	-	0.3811659	4.0938993	5.06427154	1.92E-05	0.002366465	2.774301404
LMJF_36_snoRNA0062	ncRNA	LM36Cs1C1.1	ncRNA	ena	36	942201	942298	+	-0.469562	3.4521904	-5.16354283	1.45E-05	0.002094906	3.068581156
